# Editorial: The MASLD spectrum: an emerging epidemic of cardiometabolic and extra-hepatic dimensions

**DOI:** 10.3389/fepid.2026.1893613

**Published:** 2026-06-17

**Authors:** Akash Batta, Arka De, Omesh Goyal, Aalam Sohal

**Affiliations:** 1 Department of Cardiology, Dayanand Medical College & Hospital, Ludhiana, India; 2 Department of Hepatology, Postgraduate Institute of Medical Education and Research, Chandigarh, India; 3 Department of Gastroenterology, Dayanand Medical College & Hospital, Ludhiana, India; 4 Division of Gastroenterology, Creighton University, Omaha, NE, United States

**Keywords:** cardiovascular diseases, endothelial dysfuction, inflmmation, MAFLD (metabolic dysfunction-associated fatty liver disease), MASLD, metabolic syndome, NAFLD, obesity

The transition from non-alcoholic fatty liver disease (NAFLD) to metabolic dysfunction-associated steatotic liver disease (MASLD) reflects a critical shift in our understanding of liver disease as a central theme in systemic metabolic health (1). Once considered a primarily hepatic disorder, MASLD is now recognized as a multisystem disease with profound cardiometabolic and extra-hepatic implications, including cardiovascular disease (CVD), chronic kidney disease (CKD), endocrine dysfunction, and malignancies (2–6). This paradigm shift underscores the need for a more comprehensive understanding and an integrated approach to MASLD across diverse populations. This research collection highlights this multi-dimensional impact of MASLD, extending well beyond the liver to encompass its impact on the cardiovascular, renal, nutritional, and musculoskeletal systems. As the prevalence of MASLD continues to rise globally, the integrated findings of the nine articles published in this collection provide essential insights into the epidemiology, biomarkers, and therapeutic strategies necessary to manage this complex epidemic.

The intersection of MASLD and cardiovascular health is a primary concern in type 2 diabetes (T2DM) (4). Peng et al. demonstrate a significant association between MASLD and cardiovascular autonomic neuropathy (CAN) in patients with T2DM. By evaluating heart rate variability and other autonomic indices, the study suggests that the presence of MASLD may serve as a marker for increased cardiovascular risk, specifically through autonomic dysfunction. Furthermore, the risk was directly linked to FIB-4 scores and individuals with higher FIB-4 scores had greater odds of CAN. This underscores the importance of early cardiovascular screening in MASLD patients to prevent long-term complications.

Early identification of individuals at high risk for MASLD is vital for public health and targeted screening across varied settings. Shao et al. investigated the C-reactive protein-albumin-lymphocyte (CALLY) index as a novel composite biomarker across three ethnic cohorts (US NHANES, UK Biobank, and a Chinese cohort). Their findings revealed that a lower CALLY index reflected higher inflammation, poorer nutritional status, and diminished immune competence; which in turn was strongly associated with increased MASLD prevalence. This simple, non-invasive index offers a promising tool for clinicians to stratify MASLD risk in diverse populations.

The cross talk between skeletal muscle and liver has emerged as a critical pathway in pathogenesis and progression of MASLD. A comprehensive systematic review and meta-analysis by Zuo et al. involving 185,575 participants identified sarcopenia as an independent risk factor for both MASLD and advanced liver fibrosis. In addition, sarcopenia was much more common in MASLD cohort compared to non-MASLD after accounting for all other variables. These findings highlight the importance of maintaining skeletal muscle mass as a therapeutic target in MASLD and in broader context of metabolic health.

The impact of sarcopenia on hepatic and cardiovascular outcomes in MASLD was explored by Chowdhary et al. in a large nationwide study. Sarcopenia was independently linked to hepatic decompensation, adverse cardiovascular events including heart failure, atrial fibrillation and cerebrovascular events also with overall mortality in MASLD highlighting the powerful impact of sarcopenia and its potential as a therapeutic target in MASLD.

Body composition plays a key role in MASLD development. Xu et al. utilized CT-derived measures to assess the relationship between abdominal visceral adiposity and MASLD in 7,805 Chinese adults. Their sex-stratified analysis found that visceral fat area (VFA) is more strongly associated with MASLD than traditional BMI, with distinct nonlinear patterns observed between men and women. This emphasizes the need for sex-specific approaches in assessing metabolic risk and the superiority of visceral fat quantification over generalized anthropometric measures like waist circumference and BMI.

The systemic nature of MASLD is further evidenced by its link to renal health. Wei et al. explored the association between MASLD and the risk of kidney stones using cross-sectional and cohort analyses. Their analysis suggests a positive correlation between the severity of liver steatosis and the incidence of nephrolithiasis, potentially linked through shared metabolic pathways. This finding broadens the clinical scope of MASLD management to include renal surveillance especially in high-risk individuals.

Sudhakar et al. investigated the impact of MASLD on bone mineral density (BMD) among North Indian patients with T2DM. Their study highlights a paradoxical relationship where MASLD may be associated with altered BMD, wherein early in the course of hepatic steatosis, the BMD may be increased, but as it progresses to fibrosis, this effect weans and is then associated with worsening BMD. The study supports the importance of bone health monitoring in high-risk MASLD.

Addressing the global epidemic of MASLD requires a region specific evaluation of mechanistic insights behind its pathophysiology. Basil provides a scoping-narrative review focused on the Vitamin D-MASLD-T2DM axis in Sub-Saharan Africa (SSA). The review contrasts global mechanistic insights with the unique environmental and genetic landscape of SSA, highlighting how Vitamin D deficiency may accelerate the progression of both MASLD and T2DM in this population. This underlines the necessity of localized epidemiological research and nutritional interventions for metabolic diseases.

The therapeutic landscape of MASLD is closely intertwined with management of T2DM. Naturally, there is great interest in recognizing therapies which improve both these conditions. In this context, Shao et al. explored the optimal anti-diabetic treatment for MASLD through a network meta-analysis of 21 randomized controlled trials. The study compared 15 antidiabetic agents, finding that while several agents improve liver enzymes, with ertugliflozin notably the most effective in reducing ALT and AST levels, followed by pioglitazone. This comparative analysis provides a roadmap for selecting glucose-lowering therapies that offer the greatest hepatic benefit.

The articles in this collection collectively emphasize the multisystemic nature of MASLD, requiring an integrated, multidisciplinary approach for optimal care. By highlighting novel discoveries in regards to the extra-hepatic manifestations of MASLD, this collection advances our understanding of MASLD as an emerging global health crisis. We hope this research stimulates further investigation into the holistic management of patients across the MASLD spectrum.

## References

[1] Halfon P. Évolution de la nomenclature des maladies hépatiques stéatosiques: vers un changement de paradigme. Rev Médecine Interne. (2026) 47(2):84–9. 10.1016/j.revmed.2025.12.003 41421874

[2] Zhou XD Fan QY Targher G Byrne CD Chen QF Shapiro MD. MASLD as a systemic metabolic disease: expanding the scope of cardiovascular-kidney-metabolic (CKM) syndrome. Sci China Life Sci. (2026) 69(6):1950–65. 10.1007/s11427-025-3193-9 41854965

[3] Brar AS Khanna T Sohal A Hatwal J Sharma V Singh C. Metabolic dysfunction-associated steatotic liver disease and heart failure with preserved ejection fraction: a state-of-the-art review. World J Cardiol. (2026) 18(1)18(1):111954. 10.4330/wjc.v18.i1.111954 PMID: 41607621 41607621 PMC12836017

[4] Suresh MG Mohamed S Geetha HS Prabhu S Trivedi N Ng ZC. Metabolic dysfunction-associated steatotic liver disease and type 2 diabetes: pathophysiology, diagnosis, and emerging therapeutic strategies. World J Diabetes. (2026) 17(2):113149. 10.4239/wjd.v17.i2.113149 PMID: 41696109 41696109 PMC12897526

[5] Suresh MG Mohamed S Geetha HS Prabhu S Trivedi N Mehta PD. Cardiovascular implications in metabolic dysfunction-associated steatotic liver disease (MASLD): a state-of-the-art review. Korean Circ J. (2026) 56(2):103. 10.4070/kcj.2025.0212 41371916 PMC12929038

[6] Parizad R Hatwal J Brar AS Alizadeh L Goyal MK Batta A. Interplay of childhood metabolic dysfunction-associated steatotic liver disease and obesity in the development of youth-onset type 2 diabetes. World J Clin Pediatr. (2026) 15(1):111030. 10.5409/wjcp.v15.i1.111030 PMID: 41884030 41884030 PMC13010889

